# Association of Vertical Facial Patterns With Maxillary Sinus Volume in Skeletal Class I Adults: A Cross-Sectional Cone-Beam Computed Tomographic Study

**DOI:** 10.7759/cureus.81927

**Published:** 2025-04-08

**Authors:** Paulami Bagchi, Sheetal Potnis, Akanksha Dhoke, Nikhil Daniel, Vani Jairaj, Abdul Suban A Kanna, Seema Gupta

**Affiliations:** 1 Department of Prosthodontics, D. Y. Patil Dental School, Pune, IND; 2 Department of Orthodontics, Sinhgad Dental College and Hospital, Pune, IND; 3 Department of Orthodontics, All India Institute of Medical Sciences, Jodhpur, Jodhpur, IND; 4 Department of Conservative Dentistry and Endodontics, Al-Azhar Dental College, Thodupuzha, IND; 5 Department of Orthodontics, Sri Venkateshwara Dental College and Hospital, Bengaluru, IND; 6 Department of Orthodontics, Sri Ramakrishna Dental College and Hospital, Coimbatore, IND; 7 Department of Orthodontics, Kothiwal Dental College and Research Centre, Moradabad, IND

**Keywords:** cone beam computed tomography, facial, maxillary sinus, pattern, vertical, volume

## Abstract

Introduction

The morphology of the maxillary sinus (MS) is influenced by craniofacial growth patterns, with vertical skeletal discrepancies potentially affecting MS volume. This study aimed to compare MS volume across normodivergent, hyperdivergent, and hypodivergent individuals, and to evaluate the correlation between MS volume, the mandibular plane angle, and vertical maxillary height.

Materials and methods

This retrospective, cross-sectional study included 60 adult patients (age > 18 years) with a skeletal class I pattern and fully erupted permanent dentition. The patients were divided equally into three groups (n = 20 each): normodivergent, hyperdivergent, and hypodivergent, based on the mandibular plane angle. Cone-beam computed tomography (CBCT) scans were obtained and analyzed using ITK-SNAP software for semi-automatic segmentation of MS volume (in mm³) and linear measurements of the anterior and posterior vertical maxillary heights (in mm). The bilateral MS volumes were measured independently by two calibrated examiners to ensure reliability. Statistical analysis was performed using ANOVA, Pearson correlation coefficient, and post-hoc Tukey tests (p < 0.05).

Results

The mean age was comparable across the groups (p > 0.05). No significant differences were found between the right and left MS volumes within groups, indicating bilateral symmetry. However, significant differences in MS volume and vertical maxillary height were observed across the groups (p < 0.05). Hyperdivergent patients exhibited significantly larger MS volumes and greater anterior and posterior vertical maxillary heights than hypodivergent patients. The normodivergent individuals had intermediate values. A moderate positive correlation was found between the mandibular plane angle and posterior vertical maxillary height (p = 0.016), whereas a stronger correlation was found with anterior maxillary height (p = 0.001). The MS volume showed a stronger positive correlation with vertical maxillary height than with the mandibular plane angle.

Conclusion

MS volume was significantly influenced by vertical maxillary height rather than the mandibular plane angle. Hyperdivergent individuals exhibited larger MS volumes and greater vertical maxillary heights compared to hypodivergent and normodivergent individuals. These findings highlight the role of vertical craniofacial dimensions in sinus morphology and may inform clinical decisions regarding orthodontics and maxillofacial surgery.

## Introduction

Evaluation of the maxillary sinus (MS) has become an essential component of orthodontic diagnosis and treatment planning because of its anatomical proximity to the maxillary dentition and its potential influence on various orthodontic procedures [1]. The MS is closely related to the posterior teeth and the upper alveolar process, and any changes in its dimensions or position may affect treatment outcomes [2]. Therefore, an in-depth assessment of the MS is crucial to ensure that the orthodontic treatment plan does not compromise sinus integrity or lead to complications, such as root resorption, sinus perforation, or treatment delays.

The introduction of cone-beam computed tomography (CBCT) has transformed diagnostic imaging in orthodontics. Unlike traditional two-dimensional radiographs, CBCT allows for the precise three-dimensional evaluation of craniofacial structures, including accurate measurements of MS volume [3]. The increasing adoption of CBCT in orthodontics has made it possible to study sinus anatomy in greater detail and to assess its relationship with various malocclusion patterns, particularly vertical discrepancies.

Several studies have explored the association between MS dimensions and vertical growth patterns; however, the results have been inconsistent [2,4,5,6,7]. Some studies have suggested a correlation between vertical skeletal discrepancies, such as hyperdivergent or hypodivergent growth patterns, and alterations in the MS volume [4,5]. In contrast, other studies have reported no significant relationship between sinus volume and vertical malocclusion patterns [6,7]. Factors such as sample size, age group, and the presence of mouth-breathing habits may contribute to these conflicting findings.

For instance, previous research has indicated that individuals with hyperdivergent facial types are often associated with mouth-breathing [8] and exhibit reduced sinus volume due to compromised nasal function and chronic inflammation [4]. Conversely, Wang J et al. [9] found no correlation between vertical facial patterns and changes in MS volume. These variations in research methodologies could be due to the fact that all these studies have taken variations in the mandibular plane angle as an assessment factor for vertical discrepancies. None of the studies have assessed the correlation between vertical development or position of the maxilla and volume of the MS, which complicates the interpretation of the results.

Beyond passive observation, orthodontic interventions such as rapid maxillary expansion, orthognathic surgery, molar uprighting, and canine traction have been shown to influence MS volume. Additionally, clinical considerations, such as placing mini-implants in the buccal or infrazygomatic regions, require awareness of sinus anatomy to avoid complications such as sinus perforation or implant failure. Given the clinical relevance and existing discrepancies in the literature, further research is warranted to clarify the relationship between MS volume and vertical maxillary positions. Therefore, the present study aimed to evaluate MS volume across different growth patterns using CBCT imaging. The objectives of the study were to assess if any gender or side differences exist in MS volume across the three growth patterns and to correlate the sinus volume with vertical maxillary height and mandibular plane angle. Understanding these variations is essential to guide orthodontists in creating safe and effective treatment plans that respect the sinus anatomy and optimize patient outcomes.

## Materials and methods

Study design and setting

This retrospective, cross-sectional study was conducted in the Department of Orthodontics at Kothiwal Dental College and Research Centre, Moradabad, India, following the principles of the Declaration of Helsinki. It involved the CBCT records of patients who underwent orthodontic treatment between January 2021 and August 2023. Ethical approval was obtained from our institutional review board (KDCRC/IERB/04/2024/SH03). CBCT scans were retrieved from the radiology database, and written informed consent was obtained as routine protocol from all patients before starting the study to use their records for research purposes. Patient confidentiality was maintained by anonymizing the CBCT data.

Sample size estimation

The sample size for this study was determined using G*Power software version 3.2.9 (Heinrich-Heine-Universität Düsseldorf, Düsseldorf, Germany), employing a statistical power of 80% and an alpha error of 5%. An effect size of 0.42, based on findings from a prior study by Shrestha B et al. [10], was adopted. The calculation yielded an estimated sample size of 60 patients, evenly distributed across three groups: hypodivergent, hyperdivergent, and normodivergent (20 per group).

Patient’s eligibility

Patients aged over 18 years, with a skeletal Class I pattern (normal maxillomandibular relationship assessed by an ANB angle of 2-4°, normal sagittal maxillary position assessed by an SNA angle of 82 ± 2°), fully erupted permanent dentition up to the second molars, no history of maxillofacial trauma, sinus pathology, previous surgical interventions in the maxillary region, breathing abnormalities, or habitual mouth breathing were selected for the study. Patients with congenital anomalies or syndromic conditions affecting craniofacial structures, a history of previous orthodontic treatment, or a history of sinus surgery or chronic sinusitis were excluded. The details of the ANB and SNA angles are included in Table 1.

**Table 1:** Anatomical landmarks used in the study.

S No.	Landmarks	Description
1	PNS (Posterior Nasal Spine)	The most prominent tip of the sharp bony process of the maxilla at the lower margin of the posterior nasal opening.
2	ANS (Anterior Nasal Spine)	The most prominent tip of the sharp bony process of the maxilla at the lower margin of the anterior nasal opening.
3	N (Nasion)	The most anterior point on the frontonasal suture in the midsagittal plane.
4	Point A	The most posterior midline point in the concavity between the anterior nasal spine and the prosthion.
5	Point B	The most posterior midline point in the concavity between the most superior point on the alveolar bone overlying the lower incisors and pogonion.
6	S (Sella)	Midpoint of sella turcica.
7	Go (Gonion)	The point at the intersection of tangent to the posterior border of the ramus (ramal plane) and the lower border of the mandible (mandibular plane).
8	Gn (Gnathion)	The anteroinferior point on the bony chin.
9	GoGn plane	A plane joining Go and Gn points.
10	SN plane	A plane joining S and N points.
11	ANB angle	The angle between NA line and NB line.
12	SNA angle	The angle between SN plane and NA line.
13	SN-GoGn angle (mandibular plane angle)	The angle between SN plane and GoGn plane.
14	True horizontal plane	This plane is constructed passing from the Nasion point, which is at a 7° angle to the Frankfort Horizontal plane passing through the uppermost point on external auditory meatus (Po) and the lowermost point on the bony orbit (Or).
15	Anterior vertical maxillary height (mm)	The linear distance from the true horizontal plane to the ANS by dropping a perpendicular from the true horizontal plane to the ANS.
16	Posterior vertical maxillary height (mm)	The linear distance from the true horizontal plane to the PNS by dropping a perpendicular from the true horizontal plane to the PNS.

Methodology

Sixty CBCT records were divided into three groups based on growth patterns as follows: Group 1 (n = 20) consisted of normodivergent patients with an average growth pattern (SN-GoGn angle of 27°-36°), Group 2 (n = 20) consisted of hyperdivergent patients with a vertical growth pattern (SN-GoGn angle > 36°), and Group 3 (n = 20) consisted of hypodivergent patients with a horizontal growth pattern (SN-GoGn angle < 27°). Details of the landmarks are listed in Table 1.

CBCT scans were obtained using i-CAT (Imaging Sciences Int. Inc., Pennsylvania, USA) with the following parameters: field of view (FOV) (6 x 8 cm), voxel size (0.3 mm), tube voltage (90 kVp), and tube current (5 mA). All scans were performed with the patients in the standard head position to ensure consistency. The axial section was calibrated to align with the Frankfort horizontal plane, the sagittal section to correspond to the midsagittal plane, and the coronal section to traverse the furcation of the upper first molar roots.

**Figure 1:**
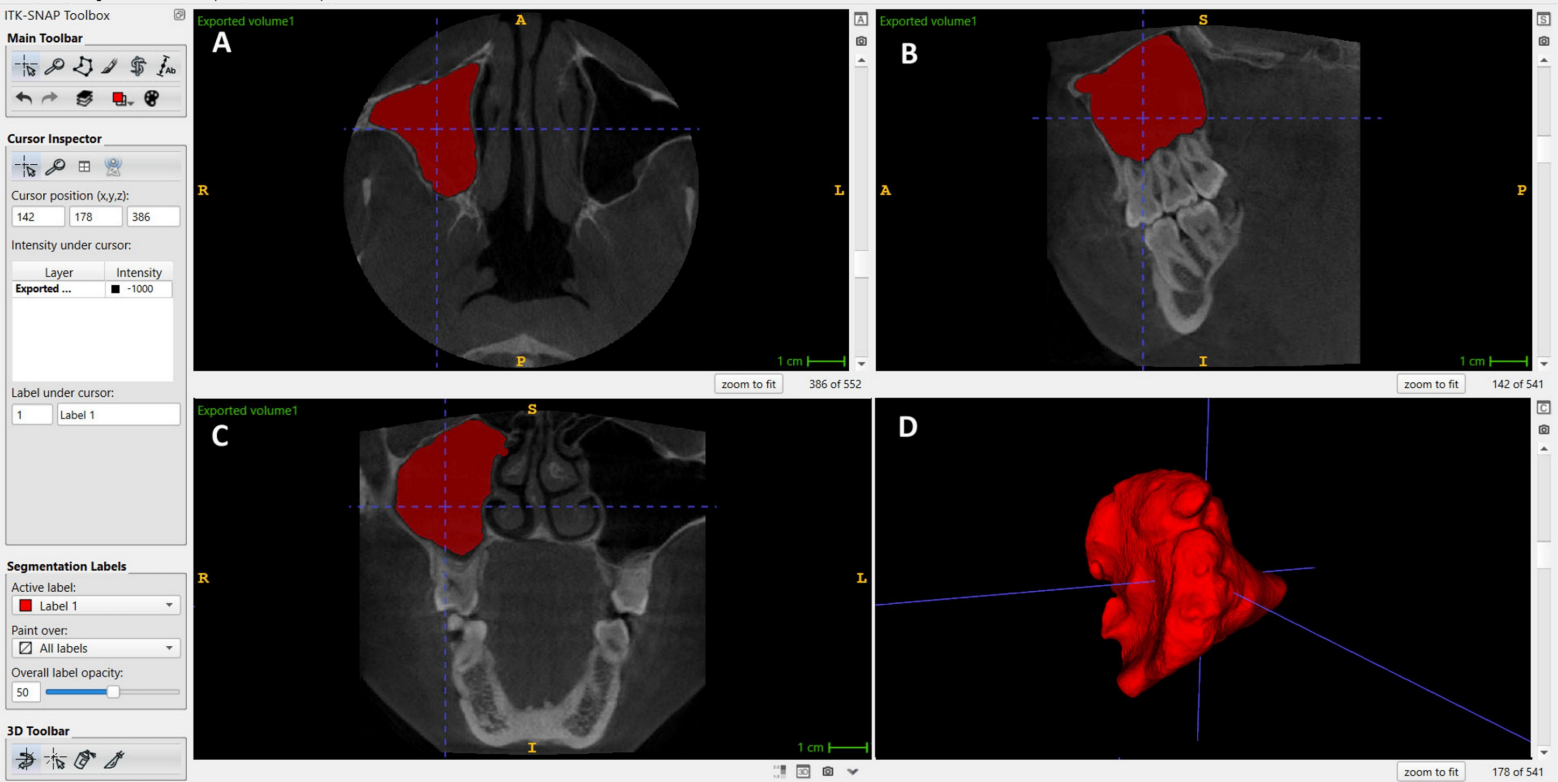
Volumetric analysis of the maxillary sinus using ITK-SNAP software: (A) Axial view of the maxillary sinus; (B) Sagittal view of the maxillary sinus; (C) Coronal view of the maxillary sinus; (D) 3D image of the right maxillary sinus volume. This image depicts the volumetric analysis of the patient's maxillary sinus, obtained as part of the study with the patient's informed consent.

The images were captured in Digital Imaging and Communications in Medicine (DICOM) format, wherein each DICOM file encapsulates a single frame with a resolution of a 512 × 512 matrix. MS volume was assessed by a calibrated prosthodontist (Bagchi P) and an orthodontist (Dhoke A) following a semi-automatic segmentation protocol using ITK-SNAP software (version 3.8, ITK-SNAP, UPenn & UNC, USA). Two demarcating lines were employed to delineate the panoramic section: a vertical line (V) and a horizontal line (H), situated at the center of the dental arch. The horizontal line was positioned at the utmost mesiodistal extension of the sinus cavity; conversely, the vertical line was ascertained from the lowest point of the sinus floor to its apex, which aligned with the boundary of the orbital floor. It extends from the medial wall of the nasal cavity to the distal wall adjacent to the tuberosity of the maxilla. The semi-automatic delineation of intracranial structures using ITK-SNAP software has been substantiated through neuroimaging research [11] and has demonstrated remarkable reproducibility [12]. Bone structures were identified with Hounsfield Unit (HU) values exceeding 700, whereas blood vessels were distinguished at HU values above 300, ensuring clear differentiation from soft tissues. The final volumetric assessment of the MS was performed and measured in mm^3^ independently by two calibrated observers (Bagchi P, Potnis S) who were blinded to group allocation to minimize interobserver bias (Figure 1).

Any discrepancies greater than 5% were reassessed and resolved by consensus. The anterior and posterior maxillary heights were measured in mm (Table 1).

Reliability assessment

To assess intra- and inter-observer reliability, 10 CBCT scans were randomly selected and remeasured after two weeks. Intraclass correlation coefficients (ICC) were calculated, with values > 0.80 considered indicative of excellent reliability.

Statistical analysis

Statistical analyses were performed using SPSS software (IBM Corp., 2013, IBM SPSS Statistics for Windows, Version 23.0, Armonk, NY, USA) by a statistician (Daniel N) who was provided with coded data. The normality of the data was assessed using the Kolmogorov-Smirnov test and corroborated by a Q-Q plot, confirming adherence to a normal distribution. Categorical variables are presented as frequencies and percentages, while continuous variables, including age, volume, and other measurements, are summarized as means and SD. One-way ANOVA was employed to compare mean values across groups. Additionally, the relationships between the variables were examined using Pearson’s correlation test. Statistical significance was established at a threshold of p < 0.05, with Bonferroni correction applied to account for multiple comparisons and to minimize the likelihood of type I errors.

## Results

The inter-observer ICC values for reliability testing were 0.84, and for intra-observer, they were 0.89, respectively, indicating excellent reliability and reproducibility. The comparison of mean age between the study groups showed no statistically significant differences across the groups. The hyperdivergent group had a mean age of 26.40 ± 4.87 years, the hypodivergent group had a mean age of 26.07 ± 4.51 years, and the normodivergent group had a mean age of 25.07 ± 4.37 years. From these findings, it can be inferred that the mean age was comparable across the groups, suggesting that age is unlikely to be a confounding factor in any of the observed differences in other variables, such as sinus volume or craniofacial measurements, among these groups. This age similarity strengthens the comparability of the groups for further analysis, ensuring that age-related developmental differences do not skew the results (Table 2).

**Table 2:** Comparison of mean age in years between study groups using an ANOVA test. P-value > 0.05 indicates non-significance. Age is represented in the form of mean and SD.

Groups	Mean	SD	95% CI for mean	F value	P-value
Hyperdivergent	26.40	4.87	23.7-29.1	0.34	0.711
Hypodivergent	26.07	4.51	23.57-28.57		
Normodivergent	25.07	4.37	22.65-27.49		

The comparison of mean sinus volumes on the right and left sides across the study groups indicated no statistically significant differences between the sides within each group (p > 0.05). For the hyperdivergent group, the mean sinus volume was 22644.33 ± 2557.29 mm³ on the right side and 22454.8 ± 2501.08 mm³ on the left side. In the hypodivergent group, the right side had a mean of 20054.93 ± 2158.0 mm³ and the left side had a mean of 19707.33 ± 1992.21 mm³. For the normodivergent group, the mean sinus volume was 21224.67 ± 2772.31 mm³ on the right and 20817.33 ± 2682.64 mm³ on the left. This suggests that sinus volume remained relatively consistent bilaterally across the groups. This bilateral symmetry may reflect a uniform developmental or anatomical pattern in sinus structure across these populations (Table 3).

**Table 3:** Comparison of mean sinus volume in mm³ on the right and left sides in study groups using independent t-tests. P-value > 0.05 indicates non-significance. Data are represented in the form of mean and SD.

Sinus volume (mm^3^)	Right		Left		T stats	P-value
	Mean	SD	Mean	SD		
Hyperdivergent	22644.33	2557.29	22454.8	2501.08	0.21	0.839
Hypodivergent	20054.93	2158.06	19707.33	1992.21	0.46	0.651
Normodivergent	21224.67	2772.31	20817.33	2682.64	0.41	0.686

Statistically significant differences were noted between the groups for mean sinus volume and mean posterior and anterior vertical maxillary heights (p < 0.05). Post-hoc analysis showed a significantly greater mean sinus volume and posterior and anterior vertical maxillary heights in the hyperdivergent group than in the hypodivergent group. Comparison of the hypodivergent and normodivergent groups revealed that the normodivergent group showed significantly greater maxillary height than the normodivergent group. These findings may indicate underlying structural or developmental differences among the groups (Table 4).

**Table 4:** Comparison of outcome variables by ANOVA followed by a post-hoc Tukey test. *p-value < 0.05 indicates significance. Data are represented in the form of means and SD. CI stands for confidence interval. 'Hyper' refers to hyperdivergent, 'Hypo' to hypodivergent, and 'Normo' to normodivergent. 'N-PNS' denotes posterior vertical maxillary height, and 'N-ANS' denotes anterior vertical maxillary height.

Variables	Groups	Mean	SD	95% CI for mean	F value	P-value	Hyper-Hypo	Hyper-Normo	Normo-Hypo
Sinus volume (mm^3^)	Hyperdivergent	22549.57	2528.67	21149.10-23950.03	4.45	0.018*	0.015*	0.288	0.633
	Hypodivergent	19881.13	2072.24	18733.45-21028.81					
	Normodivergent	21021.00	2725.54	19511.49-22530.51					
N-PNS (mm)	Hyperdivergent	49.67	3.60	47.67-51.66	14.74	0.001*	0.003*	0.257	0.001*
	Hypodivergent	44.67	2.92	43.05-46.28					
	Normodivergent	52.13	4.76	49.49-54.77					
N-ANS (mm)	Hyperdivergent	49.33	3.58	47.35-51.32	11.62	0.001*	0.001*	1.000	0.001*
	Hypodivergent	43.07	2.94	41.44-44.69					
	Normodivergent	48.80	5.03	46.01-51.59					

A moderate positive correlation was observed between the mandibular plane angle (SN-GoGn) and posterior vertical maxillary height (p = 0.016), whereas a stronger positive correlation was found between the mandibular plane angle and anterior vertical maxillary height (p = 0.001). The correlation between the anterior and posterior maxillary heights was notably high and significant. Additionally, the average sinus volume showed a moderate positive correlation with the mandibular plane angle (p = 0.012), a strong positive correlation with posterior vertical maxillary height (p = 0.001), and an even stronger positive correlation with anterior vertical maxillary height (p = 0.001). This suggests that the mandibular plane angle may have a less pronounced influence on these parameters than the anterior facial height measures (Table 5).

**Table 5:** Pearson correlation between outcome variables. *p-value < 0.05 indicates significance. N-PNS: Posterior vertical maxillary height; N-ANS: Anterior vertical maxillary height; SN-GoGn: Mandibular plane angle. Correlation strengths are categorized as follows: Weak (0.2 ≤ |r| < 0.4), Moderate (0.4 ≤ |r| < 0.6), Strong (0.6 ≤ |r| < 0.8), Very Strong (0.8 ≤ |r| ≤ 1).

Variables	SN-GoGn	N-PNS	N-ANS
N-PNS	r = 0.36	r = 0	r = 0.94
	p = 0.016*	p = 1	p = 0.001*
N-ANS	r = 0.49	r = 0.94	r = 0
	p = 0.001*	p = 0.001*	p = 1
Average sinus volume	r = 0.37	r = 0.63	r = 0.72
	p = 0.012*	p = 0.001*	p = 0.001*

In the hyperdivergent group, the average sinus volume showed a strong positive correlation with vertical maxillary height; however, a weak and non-significant negative correlation with the mandibular plane angle was observed. Similarly, in the hypodivergent group, significant positive correlations were observed with vertical maxillary height, while a moderate negative correlation with the mandibular plane angle was not statistically significant (p = 0.263). For the normodivergent group, the average sinus volume exhibited moderate positive correlations with vertical maxillary height and a non-significant positive correlation with the mandibular plane angle (p = 0.893). This suggests that in all vertical facial patterns, MS volume is correlated with vertical maxillary height rather than with the mandibular plane angle (Table 6).

**Table 6:** Pearson correlation of average sinus volume with other variables within the study groups. *p-value < 0.05 indicates significance. N-PNS: Posterior vertical maxillary height; N-ANS: Anterior vertical maxillary height; SN-GoGn: Mandibular plane angle. Correlation strengths are categorized as follows: Weak (0.2 ≤ |r| < 0.4), Moderate (0.4 ≤ |r| < 0.6), Strong (0.6 ≤ |r| < 0.8), Very Strong (0.8 ≤ |r| ≤ 1).

Group	Variable	N-PNS		N-ANS		SN-GoGn	
		r	p	r	p	r	p
Hyperdivergent	Average Sinus Volume	0.77	0.001*	0.8	0.001*	-0.13	0.633
Hypodivergent	Average Sinus Volume	0.86	0.001*	0.81	0.001*	-0.31	0.263
Normodivergent	Average Sinus Volume	0.53	0.041*	0.57	0.025*	0.04	0.893

## Discussion

The present study aimed to evaluate the relationship between MS volume and three distinct craniofacial patterns: hyperdivergent, hypodivergent, and normodivergent. The bilateral assessment of the MS volume within each group revealed no statistically significant differences between the right and left sides. This finding supports the concept of bilateral symmetry in sinus development, which is consistent with previous anatomical studies [13,14]. Such symmetry suggests a uniform genetic and developmental pattern across both sides of the maxilla, reducing concerns of asymmetry affecting volumetric analysis. It also provides a baseline of anatomical consistency for clinical practice, such as sinus augmentation procedures or surgical planning for orthognathic surgery.

Significant intergroup differences were observed in mean sinus volume, anterior vertical maxillary height, and posterior vertical maxillary height. Specifically, the hyperdivergent group exhibited significantly greater sinus volumes and maxillary heights than the hypodivergent group, whereas the normodivergent group showed significantly greater vertical maxillary heights than the hypodivergent group. These findings align with those of prior research [6,10]. The hyperdivergent pattern, typically associated with a vertically elongated face and greater lower facial height, may inherently require a more pneumatized MS to accommodate the skeletal and functional demands. This may also reflect adaptive remodeling related to vertical maxillary growth patterns [2].

Interestingly, while the general trend of larger sinus volumes in hyperdivergent individuals is consistent with prior findings, it diverges from some earlier studies reporting smaller sinus dimensions in subjects with vertical facial patterns [4]. This phenomenon may be attributable to the fact that in their research, the investigators did not omit subjects exhibiting mouth breathing; consequently, when a vertical facial pattern was correlated with mouth breathing, a diminished MS volume was observed, as evidenced in the study conducted by Tikku T et al. [8]. In the current study, the increased sinus volume in the hyperdivergent group could be attributed to a more pronounced vertical development of the maxillary complex and the non-inclusion of patients with nasal abnormalities, supporting the positive correlations observed between sinus volume and vertical maxillary height.

Correlation analyses further underscored these relationships. A moderate-to-strong positive correlation between sinus volume and both anterior and posterior vertical maxillary heights was noted across all groups. These findings support the hypothesis that MS size is more closely related to vertical maxillary dimensions than to the mandibular plane angle alone. In particular, the anterior vertical maxillary height demonstrated a stronger association with sinus volume than the posterior height. These results are in line with the observations of previous studies suggesting that sinus pneumatization may follow the pattern of vertical maxillary growth, particularly in the anterior region, where structural support for the nasal cavity and midface is critical [15,16].

Interestingly, the mandibular plane angle exhibited weaker and non-significant correlations with sinus volume across the individual groups, suggesting its limited role in influencing sinus development when analyzed in isolation. This may indicate that while the mandibular plane angle reflects the overall vertical facial divergence, it is not a direct determinant of MS volume. Rather, its influence may be secondary and mediated through its relationship with vertical maxillary dimensions. Prior literature also points to the mandibular plane angle as a marker of skeletal pattern rather than a functional driver of sinus pneumatization [17].

These findings have several important implications. The relationship between sinus volume and vertical maxillary height suggests that practitioners should consider the vertical skeletal pattern when planning maxillofacial procedures such as sinus lifts, implant placements, or corrective jaw surgeries. In hyperdivergent individuals with increased maxillary height and larger sinus volume, surgical planning should account for potentially deeper sinus cavities and altered spatial relationships within the midface. Conversely, hypodivergent patients with reduced sinus volumes and maxillary heights may present with different anatomical challenges, including a relatively flatter palatal vault and less pneumatized MS [18].

Furthermore, the high correlation between anterior and posterior maxillary heights in this study reinforces the interconnected nature of vertical skeletal components. Vertical discrepancies in maxillary height may influence midfacial projections and occlusal plane inclinations, which are often implicated in dentofacial deformities and occlusal disorders. Therefore, sinus development, maxillary height, and mandibular plane orientation should be assessed during the diagnosis and treatment planning of craniofacial anomalies.

Limitations of the study

Despite the valuable insights provided by this study, some limitations should be acknowledged. First, the study's design was retrospective and cross-sectional, which inherently limits the ability to establish causal relationships between variables such as facial growth patterns and MS volume. A longitudinal study would better elucidate the developmental trajectories of sinus volume and craniofacial dimensions. Second, although statistically justified, the sample size was relatively modest, which may limit the generalizability of the findings to broader populations with different ethnic, genetic, and environmental backgrounds. Additionally, only skeletal Class I patients were included, thereby excluding other sagittal skeletal patterns (such as Class II or Class III), which could also influence sinus development and craniofacial structure. Furthermore, factors such as breathing habits, environmental influences, or mild subclinical sinus pathology, which could potentially affect MS morphology, were not assessed. Lastly, while efforts were made to reduce inter-observer variability through calibration, some degree of measurement bias cannot be entirely excluded.

## Conclusions

This study demonstrated that MS volume and vertical maxillary height were significantly greater in hyperdivergent individuals compared to their hypodivergent and normodivergent counterparts. Additionally, consistent bilateral symmetry in sinus volume was observed across all facial growth patterns, suggesting a stable anatomical pattern in these populations. Importantly, MS volume showed a stronger correlation with anterior and posterior vertical maxillary heights than with the mandibular plane angle, indicating that vertical maxillary dimensions are more influential on sinus morphology than mandibular divergence alone. These findings suggest that clinicians should consider vertical facial patterns and associated maxillary heights when evaluating sinus anatomy, particularly in treatment planning for sinus-related procedures, such as orthodontic interventions, maxillary surgeries, or sinus augmentation procedures. Future studies with larger sample sizes, the inclusion of diverse skeletal patterns, and longitudinal data are warranted to validate these findings and further explore the interplay between craniofacial structures and MS development.
